# Evaluation of the Clinical Effectiveness of Oseltamivir for Influenza Treatment in Children

**DOI:** 10.3389/fphar.2022.849545

**Published:** 2022-04-06

**Authors:** Jianru Qin, Jilei Lin, Xiangfei Zhang, Shuhua Yuan, Chiyu Zhang, Yong Yin

**Affiliations:** ^1^ NMPA Key Laboratory for Research and Evaluation of Innovative Drug, College of Life Sciences, Henan Normal University, Xinxiang, China; ^2^ Department of Respiratory, Shanghai Children’s Medical Center Affiliated to Shanghai Jiao Tong University School of Medicine, Shanghai, China; ^3^ Shanghai Public Health Clinical Center, Fudan University, Shanghai, China

**Keywords:** clinical effectiveness, Oseltamivir, treatment, influenza, children

## Abstract

**Objective:** To estimate the clinical effectiveness of oseltamivir in children with different subtypes of influenza virus infection.

**Methods:** A total of 998 children with acute respiratory infection were enrolled from January to March 2018, and were divided into influenza A, influenza B, influenza A + B, and non-influenza infection (IV-negative) groups. Influenza-like symptoms and duration of fever were evaluated and compared between oseltamivir-treated and non-treated groups.

**Results:** There were no significant differences in the reduction in total febrile period and duration of fever from the onset of therapy between the oseltamivir treated and non-treated children infected with influenza A (*p* = 0.6885 for total febrile period and 0.7904 for the duration of fever from the onset of treatment), influenza B (*p* = 0.1462 and 0.1966), influenza A + B (*p* = 0.5568 and 0.9320), and IV-negative (*p* = 0.7631 and 0.4655). The duration of fever in children received oseltamivir therapy within 48 h was not significantly shorter than that beyond 48 h (*p* > 0.05). Additionally, percentages and severities of influenza-like symptoms, including headache, myalgia, fatigue, bellyache, vomiting, diarrhea, sore throat, cough, and coryza were not decreased and alleviated after treatment of oseltamivir.

**Conclusion:** Oseltamivir treatment does not significantly shorten the duration of fever, nor does it significantly relieve influenza-like symptoms in children with infection of influenza.

## Introduction

Seasonal influenza epidemics, caused by the influenza A (H1N1 and H3N2) and influenza B viruses pose a great threat to the health of children each year (Lytras et al., 2019; Mott et al., 2021). The annual incidence rate of seasonal influenza can be up to 30% in the entire pediatric population (Esposito and Principi, 2016). Moreover, seasonal influenza infection is usually characterized by severe clinical manifestations and complications, such as rhinosinusitis, pneumonia, myocarditis, encephalitis, gastroenteritis, acute otitis media, and acute respiratory distress syndrome, resulting in considerably high hospitalization and mortality rates in children (Neuzil et al., 2000; Ferdinands et al., 2011; Antonova et al., 2012; Asseri et al., 2021; RothDiPrinzioFisher, 2021).

Currently, there are only two classes of specific antiviral drugs that have been approved for the treatment of influenza virus infections: M2-ion channel inhibitors and neuraminidase inhibitors (NAIs) (YipSelim et al., 2018). M2-ion channel inhibitors are only effective against influenza A virus, and are rarely recommended for clinical use because most influenza strains have developed resistance to them (Toledo-Rueda et al., 2018; Vorobjev, 2021). Hence, NAIs, which include oseltamivir, zanamivir, lanimamivir, and peramivir, are the only available anti-influenza virus drugs (Zwillenberg et al., 2021). Oseltamivir is the most widely prescribed NAI and has been extensively used in the prophylaxis and treatment of both influenza A and influenza B virus infections (Davies, 2010). Additionally, oseltamivir is the most commonly used drug in children (Esposito and Principi, 2016).

Despite the fact that influenza poses a great burden on children and oseltamivir is widely used for the treatment of influenza in this population, there are few studies on the clinical efficacy of oseltamivir in children compared to adults, or on the effectiveness of oseltamivir in infection caused by the different subtypes of the influenza virus. In this study, we analyzed a large number of children who were diagnosed with influenza A, influenza B, co-infection with influenza A and influenza B (designated as influenza A + B), and non-infection with influenza A or B (designated as IV-negative) using an influenza antigen detection test kit, and who were treated with oseltamivir or not. The intensity of symptoms and duration of fever were compared to assess the efficacy of oseltamivir treatment.

## Methods

### Study Design and Participants

The observational real-world study was conducted in Shanghai Children’s Medical Center, a 1000-bed tertiary teaching hospital in Shanghai, China. Patients were enrolled from January 2018 to March 2018.

The criteria for enrollment in this study were formulated in accordance with the guidelines for the diagnosis and treatment of influenza issued by the Ministry of Health of China in 2011, and by the respiratory group of the Chinese Academy of Pediatrics in 2015. The inclusion criteria were children aged 0 months to 16 years with influenza-like illness (such as fever, acute upper respiratory symptoms or other systemic symptoms) or a positive rapid influenza test result who visited the outpatient department or emergency department of the hospital. No exclusion criteria were used for enrollment. Detailed patient information, including the courses of treatment, and influenza-like symptoms, such as fever, cough, coryza (sneezing, runny nose, nasal congestion), sore throat, headache, myalgia, fatigue, bellyache, vomiting, and diarrhea was recorded. Moreover, the body temperature of the children should have been measured at least two times per day (8:00 and 20:00, body temperature <37.0°C was considered afebrile). The severity of cough and coryza was divided into four degrees: absent, mild, moderate, and severe. The white blood cell (WBC) count, blood platelet count (BPC), C-reactive protein (CRP), hemoglobin (Hb), and percentage of neutrophils (N%) were measured by routine peripheral blood examination before administration of any treatment.

### Influenza Antigen Detection Test

Influenza antigens were detected in nasal and laryngeal specimens. A colloidal gold immunochromatographic assay (Wondfo Co., Ltd.) was performed for the rapid detection of influenza virus A and B antigens. This method uses highly unique monoclonal antibodies against the influenza A and B viruses. When the antigen concentration of the sample to be tested is higher than the minimum, it forms a complex with the labeled antibody, moves, and is captured by the monoclonal antibody of influenza virus nucleoprotein under the action of chromatography to form a red reaction line. The test is completed within 15–20 min. The accuracy of the colloidal gold immunochromatographic assay with virus isolation, which is considered the gold standard for influenza detection, was 90.24–92.09% for influenza A, 98.36%–99.59% for influenza B, and 87.39–90.70% for influenza A + B.

Oseltamivir is the only approved anti-influenza drug for the treatment of children. After a comprehensive analysis of the children’s symptoms, age, preference, and presence of chronic diseases, children weighing <37.5 kg were treated with oral oseltamivir at a dose of 2 mg/kg, and children weighing ≥37.5 kg were treated with oral oseltamivir at a dose of 75 mg twice a day for five consecutive days. After the initial treatment, a follow-up clinical examination was conducted on days 3 and 10 to monitor the disease progression.

### Statistical Analysis

Statistical analyses were performed using SPSS and GraphPad Prism version 6. Chi-square test, unpaired Student’s t-test, and one-way ANOVA followed by Tukey’s multiple comparison test were used for statistical comparisons and statistical analysis. Statistical differences between the two groups are indicated by ∗(*p* < 0.05), ∗∗(*p* < 0.01), ∗∗∗(*p* < 0.001), ∗∗∗∗(*p* < 0.0001).

## Results

### Characteristics of the Patients

A total of 998 children with the mean age of 4.82 ± 2.73 years were enrolled in the present study. Of these, 325 were infected with influenza A virus (266 were treated with oseltamivir and 59 were not), 232 were infected with influenza B virus (172 were treated with oseltamivir and 60 were not), 51 were co-infected with influenza A and influenza B viruses (45 were treated with oseltamivir and six were not), and 390 were IV-negative (23 were treated with oseltamivir, 365 were not and two were off the record for the information of oseltamivir treatment was not available) according to the results of influenza antigen detection tests. The demographic characteristics of the children are summarized in table 1. The sex, average time until treatment, N% and BPC did not differ significantly among the four groups, with *p* values of 0.142, 0.6714, 0.2410, and 0.1135, respectively (Table 1). However, significant differences were observed in the age, peak body temperature (Supplementary Figure S1), peak body temperature over the past 24 h, WBC count, and Hb among the four groups, with *p* values of 0.0002, 0.0000, 0.0000, 0.0023, and 0.0163, respectively (Table 1).

**TABLE 1:** Characteristics of children patients enrolled in this study.

		Influenza A	Influenza B	Influenza A + B	IV-Negative	*p* Value
Total No. of patients		325	232	51	390	-
Gender	No. of male	184	120	35	208	0.142
	No. of female	140	112	16	178	
Age (years, Mean ± SD)		4.50 ± 2.57	5.45 ± 2.66	4.16 ± 3.14	4.80 ± 2.77	0.0002
Time until treatment (hours, Mean ± SD)		62.28 ± 42.05	61.79 ± 36.15	61.71 ± 40.95	65.92 ± 50.25	0.6714
Temperature	Peak body temperature (°C, Mean ± SD)	39.63 ± 0.70	39.38 ± 0.60	39.63 ± 0.66	39.34 ± 0.68	0.0000
	Peak body temperature over the past 24 h	39.37 ± 0.88	39.16 ± 0.68	39.37 ± 0.86	39.08 ± 0.79	0.0000
Routine peripheral blood examination	WBC (*10^9^/L)	6.98 ± 3.75	6.62 ± 3.24	7.12 ± 2.74	7.90 ± 5.20	0.0023
	N%	58.89 ± 18.66	56.52 ± 15.42	55.67 ± 17.56	56.49 ± 17.28	0.2410
	HB (g/L)	122.95 ± 10.74	125.66 ± 10.28	121.69 ± 11.41	125.05 ± 10.56	0.0163
	BPC (*10^9^/L)	195.15 ± 76.67	194.07 ± 57.93	212.60 ± 79.92	205.70 ± 76.24	0.1135

Note: The genders of one child in influenza A and four children in IV-negative were off record. -: not applicable.

### Duration of Fever

Inconsistent with the results of previous studies that oseltamivir is effective in shortening the duration of fever after the onset of treatment in influenza (Kawai et al., 2006; Groeneveld et al., 2020), our results showed that though the total febrile periods in children infected with influenza A or influenza B treated with oseltamivir were shorter than those not treated with oseltamivir, the differences were not statistically significant (*p* = 0.6885 for influenza A and 0.1462 for influenza B). Total febrile periods in patients of infected with A + B or IV-negative treated with oseltamivir were higher than those in patients not treated with oseltamivir (*p* = 0.5568 for influenza A + B, and 0.7631 for IV-negative) (Table 2). Furthermore, the total febrile period in influenza A treated with oseltamivir was not shorter than that in influenza B treated with oseltamivir (*p* = 0.6457), nor was it shorter in influenza A compared to influenza A + B (*p* = 0.3168), and in influenza B compared to influenza A + B (*p* = 0.3817).

**TABLE 2:** Comparison for the duration of fever of oseltamivir treated and non-treated children infected with different subtypes of influenza.

		Influenza A	Influenza B	Influenza A + B	IV-Negative	*p* Value
Total febrile period	Oseltamivir treated	110.29 ± 48.84 (220)	108.06 ± 38.67 (143)	101.84 ± 37.60 (37)	118.80 ± 51.74 (20)	0.5453
	Oseltamivir non-treated	113.28 ± 41.30 (50)	117.74 ± 47.61 (53)	91.20 ± 38.40 (5)	114.97 ± 55.21 (308)	0.7499
	*p* value	0.6885	0.1462	0.5568	0.7631	-
Duration of fever from onset of treatment	Oseltamivir treated	47.89 ± 26.34 (220)	47.33 ± 23.91 (143)	44.11 ± 22.67 (37)	44.40 ± 24.33 (20)	0.8069
	Oseltamivir non-treated	48.96 ± 22.49 (50)	52.53 ± 27.60 (53)	43.20 ± 17.96 (5)	49.48 ± 30.45 (308)	0.8502
	*p* value	0.7904	0.1966	0.9320	0.4655	-

Note: Data are Mean h ± SD (No. of patients)-: not applicable.

The duration of fever from the commencement of oseltamivir therapy was also compared to assess the effectiveness of oseltamivir. There was no statistically significant difference between the oseltamivir treatment and non-treatment groups, with *p* values of 0.7904 (influenza A), 0.1966 (influenza B), 0.9320 (influenza A + B), and 0.4655 (IV-negative) (Table 2). The differences in the fever duration from the start of oseltamivir treatment between influenza A and influenza B (*p* = 0.8376), influenza A and influenza A + B (*p* = 0.4114), influenza B and influenza A + B (*p* = 0.4617) were not statistically significant.

Overall, oseltamivir did not shorten the duration of fever, regardless of the onset of illness or the onset of treatment.

### Time From the Onset of Symptoms to the Start of Oseltamivir Treatment

Previous studies have shown that oral oseltamivir treatment should be started within 48 h of symptoms onset (Groeneveld et al., 2020). Hence, we analyzed the effect of the time from the onset of therapy on the effectiveness of oseltamivir. As shown in Table 3, regardless of whether treatment was initiated within or beyond 48 h, oseltamivir did not shorten the fever duration (*p* = 0.1186–0.9003 for the total febrile period and *p* = 0.0964–0.7716 for fever duration from the onset of treatment). Except the duration of fever from the onset of treatment when treatment was started beyond 48 h in children infected with influenza A (the average duration in oseltamivir treated children was 7.5 h longer than that in non-treated children), oseltamivir treatment reduced the fever period by 2–10 h in children, but there were no statistical differences (Table 3).

**TABLE 3:** Effect of time to start of oseltamivir administration on duration of fever.

			Influenza A	Influenza B	Influenza A + B	IV-Negative	*p* Value
Total febrile period	≤48 h	Oseltamivir treated	86.18 ± 24.94 (127)	85.16 ± 28.27 (80)	85.44 ± 19.30 (25)	81.60 ± 30.74 (10)	0.9547
		Oseltamivir non-treated	93.68 ± 18.65 (31)	91.71 ± 24.89 (28)	78.00 ± 31.17 (4)	86.62 ± 34.42 (178)	0.5529
		*p* value	0.1186	0.2796	0.5153	0.6525	-
	>48 h	Oseltamivir treated	143.23 ± 54.01 (93)	137.14 ± 29.45 (63)	136.00 ± 43.08 (12)	156.00 ± 40.52 (10)	0.5911
		Oseltamivir non-treated	145.26 ± 47.67 (19)	146.88 ± 50.03 (25)	-(1)	153.78 ± 54.70 (130)	0.7134
		*p* value	0.8794	0.3706	-	0.9003	-
Duration of fever from onset of treatment	≤48 h	Oseltamivir treated	50.65 ± 22.18 (127)	50.70 ± 24.88 (80)	46.08 ± 19.10 (25)	45.60 ± 25.06 (10)	0.7356
		Oseltamivir non-treated	56.52 ± 15.60 (31)	52.29 ± 24.89 (28)	42.00 ± 19.90 (4)	52.04 ± 32.43 (178)	0.7772
		*p* value	0.1664	0.7716	0.6961	0.5379	-
	>48 h	Oseltamivir treated	44.13 ± 30.71 (93)	43.05 ± 21.87 (63)	40.00 ± 28.28 (12)	43.20 ± 23.52 (10)	0.9668
		Oseltamivir non-treated	36.63 ± 26.25 (19)	52.80 ± 30.36 (25)	-(1)	45.97 ± 27.13 (130)	0.1560
		*p* value	0.3233	0.0964	-	0.7542	-

Data are Mean h ± SD (No. of patients).-: not applicable.

Although the percentages of children infected with influenza A and influenza B afebrile within 24 and 48 h from onset of oseltamivir treatment were higher than those of oseltamivir non-treatment, no significant difference was observed. The percentage of body temperature of children treated with oseltamivir become normal within 24 h were up to 27.27–32.43% compared to 22.00–29.22% in oseltamivir non-treated groups (Table 4). Within 48 h, the percentages of children afebrile with oseltamivir treatment were 69.23–72.97%, which were higher than 56.6–64.00% of the oseltamivir non-treated groups (Table 4).

**TABLE 4:** Percentage of patients afebrile within 24 and 48 h from onset of treatment.

		Influenza A	Influenza B	Influenza A + B	IV-Negative
24	Oseltamivir treated	27.27% (60)	30.77% (44)	32.43% (12)	30.00% (6)
	Oseltamivir non-treated	22.00% (11)	26.42% (14)	20.00% (1)	29.22% (90)
48	Oseltamivir treated	71.36% (60 + 97)	69.23% (44 + 55)	72.97% (12 + 15)	70.00% (6 + 8)
	Oseltamivir non-treated	64.00% (11 + 21)	56.60% (14 + 16)	60.00% (1 + 2)	62.66% (90 + 103)

### Symptoms of Influenza-Like Illness

**FIGURE 1:**
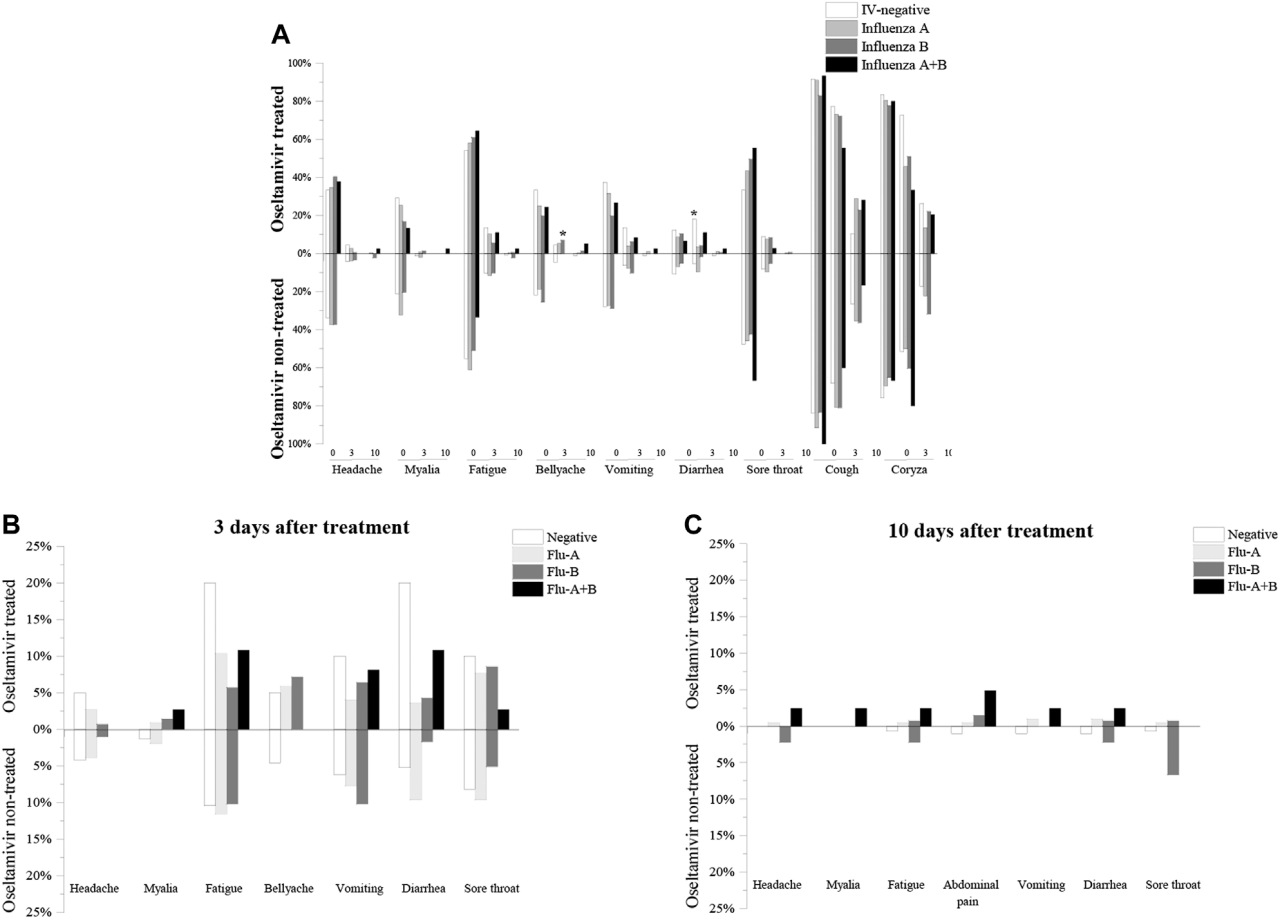
Comparision of influenza-like symptoms of oseltamivir treated and non-treated groups. **(A)**: Percentage of influenza-like symptoms of oseltamivir treated and non-treated groups before treatment. **(B)**: Percentage of influenza-like symptoms of oseltamivir treated and untreated groups after 3 days of treatment. **(C)**: Percentage of influenza-like symptoms of oseltamivir treated and untreated groups after 10 days of treatment.

Coryza and cough were the most common influenza-like symptoms, followed by the less common symptoms of fatigue, sore throat, headache, vomiting, bellyache, and myalgia, with diarrhea being the most common (Supplementary Figure S2A). The percentage of each symptom showed a decreasing trend over time (0 d, 3 d, and 10 days after treatment), and there were almost no symptoms at 10 days after treatment except for cough and coryza (Supplementary Figures S2A–C), indicating that influenza was in the process of resolution. Comparison of the percentage of symptoms in patients treated and not treated with oseltamivir revealed that there were no significant differences between the two groups, with the exception of diarrhea 3 days after treatment in the IV-negative group (oseltamivir treated vs. oseltamivir non-treated was 4/18 vs. 16/290, *p* = 0.0216), which indicates that oseltamivir treatment may not relieve influenza-like symptoms; conversely, it may have exacerbated some symptoms in IV-negative children (Figure 1).

**FIGURE 2:**
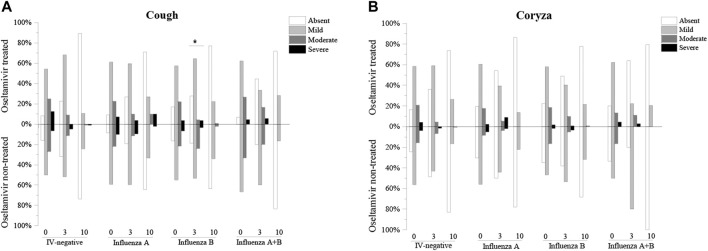
Comparision of the severities of cough and coryza of oseltamivir treated and non-treated groups. **(A)**: Comparision of the severities of cough of oseltamivir treated and non-treated groups. **(B)**: Comparision of the severities of coryza of oseltamivir treated and non-treated groups.

Moreover, the severity of cough and coryza was divided into four degrees. The rate of moderate and severe coryza tended to show a decrease compared to absent and mild coryza, which tended to increase with time. There was a significant difference in the cough severity after 3 days of influenza B treatment between the oseltamivir treated group and the oseltamivir non-treated group (number of cases of absent, mild, moderate, and severe cough in the oseltamivir treated group were 39, 91, 6, and 5, respectively, whereas the number of cases of absent, mild, moderate, and severe cough in the non-treated oseltamivir were 11, 31, 14, and 2, respectively, *p* = 0.0000) (Figure 2). In addition, no differences were found in the severity of cough and coryza between the oseltamivir treated and non-treated groups (Figure 2).

### Adverse Effects

Although oseltamivir is generally well tolerated, adverse effects are reported to be relatively common in patients, especially in infants and young children (Rath et al., 2015). The proportion of adverse events in children treated with oseltamivir (51/506) was significantly higher (*p* = 0.0000) than in those not treated with oseltamivir (14/490), which was consistent with previous studies (Jefferson et al., 2014). The proportions of adverse effects induced by oseltamivir were 4/23 (17.39%) in the IV-negative group, 21/266 (7.89%) in influenza A infection, 19/172 (11.05%) in influenza B infection, and 7/45 (15.56%) in influenza A + B infection, whereas in the non-treated children, adverse effects were observed in proportions of 3/365 (0.82%), 5/59 (8.47%), 6/60 (10.00%), and 0/6, respectively. There was a significant difference (*p* = 0.000) in adverse events between the oseltamivir treated (4/23) and non-treated (3/365) children in the IV-negative group, but no significant differences were found in children with influenza A (oseltamivir treated vs. oseltamivir non-treated was 21/266 vs. 5/59, *p* = 0.9071), influenza B (19/172 vs. 6/60, *p* = 0.8219), and A + B (7/45 vs. 0/6, *p* = 0.6828). Nausea (*n* = 31) and bellyache (*n* = 15) were the most common adverse effects reported in children treated with oseltamivir, and all the adverse events were mild to moderate. Other adverse effects induced by oseltamivir, including dizziness, nosebleed, stomachache, and poor appetite, were less common. No neuropsychiatric symptoms were observed, and treatment was not prematurely withdrawn for adverse events in any patient (Table 5).

**TABLE 5:** Adverse effects after treatment.

	Influenza A	Influenza B	Influenza A + B	IV-Negative
Oseltamivir treated				
Total	7.89% (21)	11.05% (19)	15.56% (7)	17.39% (4)
Nausea	4.14% (11)	6.97% (12)	13.33% (6)	8.70% (2)
Bellyache	2.63% (7)	3.49% (6)	0	8.70% (2)
Others	1.13% (3)	0.58% (1)	2.22% (1)	0
Oseltamivir non-treated				
Total	8.47% (5)	10.00% (6)	0	0.82% (3)
Nausea	5.08% (3)	3.33% (2)	0	0.27% (1)
Bellyache	3.39% (2)	3.33% (2)	0	0.27% (1)
Others	0	3.33% (2)	0	0.27% (1)

Other side effects including dizziness, nosebleed, stomachache and poor appetite. Data are percentage (No. of patients).

## Discussion

Although oseltamivir demonstrated excellent safety and tolerability *in vivo* (Davies, 2010), in recent years, many experts have raised doubts and controversies regarding the efficacy of oseltamivir treatment in patients with influenza, especially children. The most important reason is that there are significantly fewer studies in children than in adults, let alone studies to compare the effectiveness of oseltamivir in the treatment of different subtypes of influenza. In addition, there were some design defects in observational studies on the effect of oseltamivir, such as the number of children being too small for the analysis or the criteria of effectiveness being different for comparison. Furthermore, influenza in children can lead to multiple complications, and whether the children develop a chronic illness remains unknown. In the present study, we enrolled a large number of children who were diagnosed with an influenza-like illness, and they were divided into four groups: influenza A, influenza B, influenza A + B, and IV-negative based on the results of the influenza antigen detection test kit for the identification of the subtypes of influenza. The clinical effectiveness of oseltamivir in the treatment of different subtypes of influenza was evaluated and compared. As an observational study on the efficacy of oseltamivir, this research not only provides a large amount of data on influenza in children, but also has important implications for the management of influenza B, whose epidemiology and impact on public health are less understood and often underestimated.

The limited clinical effectiveness of oseltamivir in the treatment of influenza has been reported previously. Muthuri et al. pointed out that although NAIs treatment was associated with reduced mortality in adult patients infected with influenza A H1N1pdm09, the mortality risk was not reduced in pediatric patients (Muthuri et al., 2014). Santtu et al. demonstrated that oseltamivir could not decrease the incidence of acute otitis media even after starting therapy within 24 h. In addition, oseltamivir was been demonstrated no efficacy against influenza B infection in children (Heinonen et al., 2010). Wang et al. summarized that treatment of children with oseltamivir resulted in a reduction in the duration of the illness and alleviation of symptoms, although it did not achieve statistical significance (Wang et al., 2012). There has been much debate surrounding the efficacy of oseltamivir, including the lack of significant therapeutic effect on the incidence of pneumonia, sinusitis, bronchitis, and otitis media (Heinonen et al., 2010; Toovey et al., 2012; Wang et al., 2012; Jefferson et al., 2014; Muthuri et al., 2014). In accordance with the previous studies, although there were no significant differences between the oseltamivir treated and non-treated groups in the present study, oseltamivir therapy showed a trend towards reducing the duration of fever in children infected with influenza A and influenza B (no. of children not treated with oseltamivir was five, which is too small for statistical analysis). In terms of the symptom relieving effects, oseltamivir treatment and non-treatment groups were comparable. Whether the safety of oseltamivir treatment is greater than its effectiveness has also been questioned. Nguyen reported the case of a 14-year-old girl who was treated with oseltamivir, and developed systemic lupus erythematosus, systemic vasculitis, chronic pancreatitis, and eventually died of the complications (Nguyen et al., 2010). In addition, psychiatric side effects after oseltamivir treatment are more common in children than in adults (JhonKimKangKimLeeKim, 2021). Influenza viruses mutate easily, and there is little treatment for oseltamivir-resistant influenza (HanpaiboolLeelawiwatTakahashiRungrotmongkol, 2021; Macesic et al., 2021).

Considering the fact that oseltamivir has no significant effect on the treatment of influenza in the clinic and the high rate of side effects in children, it is important to identify suitable antiviral alternatives. Favipiravir (T-705), an inhibitor of viral RNA polymerase, has been proven to be effective in the treatment of influenza viruses, including NAI-resistant variants, and is also a potential drug for treating Ebola virus disease virus (EVD) and severe acute respiratory syndrome coronavirus type 2 (SARS-cov-2) (FangHuangLiChengTanLiu, 2020; DziedziejkoPawlik, 2021). Baloxavir marboxil (baloxavir), a novel influenza cap-dependent inhibitor of endonuclease-selective polymerase acidic protein, has shown clinical efficacy in rapidly reducing the viral load, shortening the duration of fever, and relieving symptoms (Chong et al., 2021; Portsmouth et al., 2021). In addition, compared to the ineffectiveness of NAIs, the efficacy of single and combined use of favipiravir was excellent *in vivo* (Imai et al., 2017; Wang et al., 2020). However, these antiviral drugs are still approved for restricted use or clinical trials in some countries. In addition to anti-influenza drugs, the usefulness of influenza vaccines cannot be overemphasized. Influenza vaccination in susceptible children has been shown to be an effective measure for preventing influenza virus infection, but a “Universal” vaccine with a broad spectrum of protection needs to be developed due to rapid viral mutations (Rolfes et al., 2019; Niang et al., 2021).

This study has some limitations, which must be noted. First, it was performed in a general practice setting instead of in the context of a rigorous clinical protocol. Second, the number of children infected with the different subtypes of the influenza virus varied greatly, as did the number of oseltamivir treated and non-treated children, especially the number of non-treated children infected with influenza A + B, which was too small for statistical analysis. Third, the administration of oseltamivir within 48 h referred to less than 48 h from the onset of fever, instead of the onset of symptoms. It is difficult for infants and young children (aged <2 years) to express the onset of symptoms. Hence, fever, as an important indicator of influenza, can be detected by the measurement of temperature and is much more accurate and convenient to record. Nonetheless, therapy within 48 h from the onset of fever does not mean it was within 48 h from the onset of symptoms, unless fever was the first symptom. In addition, although a lot of efforts had been made to collect children raw data, there are still some gaps. For example, we collected 325 children infected with influenza A, but in terms of statistics of duration of fever, only 270 children were counted, partly because several children were afebrile (*n* = 1), and partly because of the lack of original data (*n* = 54). And we displayed the number to statistics in brackets in each table to solve this problem. It is worth mentioning that the data presented here showed that oseltamivir has no significant effect on relieving influenza-like symptoms, instead of in treating influenza. In this study, we did not perform assay on influenza virus isolation or virus resistance. Hence, the role of oseltamivir in reducing viral particle release is hard to clarify in this study. Data of children from 2018 influenza epidemic season was analyzed in this study, more data and other influenza epidemic seasons should be collected for further studies to verify the conclusion.

In conclusion, the evidence presented in this research shows that the duration of fever in children with influenza virus infection was not reduced by the administration of oseltamivir. Moreover, influenza-like symptoms were not relieved, and the severity of cough and coryza was not improved by administering oseltamivir.

## Data Availability

The original contributions presented in the study are included in the article/Supplementary Material, further inquiries can be directed to the corresponding author.

## Supplementary Material

Supplementary Figure S1 Peak body temperatures of children with infection of influenza A, influenza B, influenza A+B, and IV negative.

Supplementary Figure S2 Percentage of influenza-like symptoms before and after oseltamivir treatment. **(A)**: Percentage of influenza like symptoms before oseltamivir treatment. **(B)**: Percentage of influenza like symptoms after 3 days of oseltamivir treatment. **(C)**: Percentage of influenza like symptoms after 10 days of oseltamivir treatment.

Supplementary Figure S3 The severity of cough and coryza before and after oseltamivir treatment. **(A)**: Cough severity before and after 3 and 10 days of oseltamivir treatment. **(B)**: Coryza severity before and after 3 and 10 days of oseltamivir treatment.
