# Supplementary figures and images for: Evaluation of the Clinical Effectiveness of Oseltamivir for Influenza Treatment in Children
Source: Front Pharmacol. 2022 Apr 6;13:849545. doi: 10.3389/fphar.2022.849545 (PMC9020783; doi:10.3389/fphar.2022.849545)

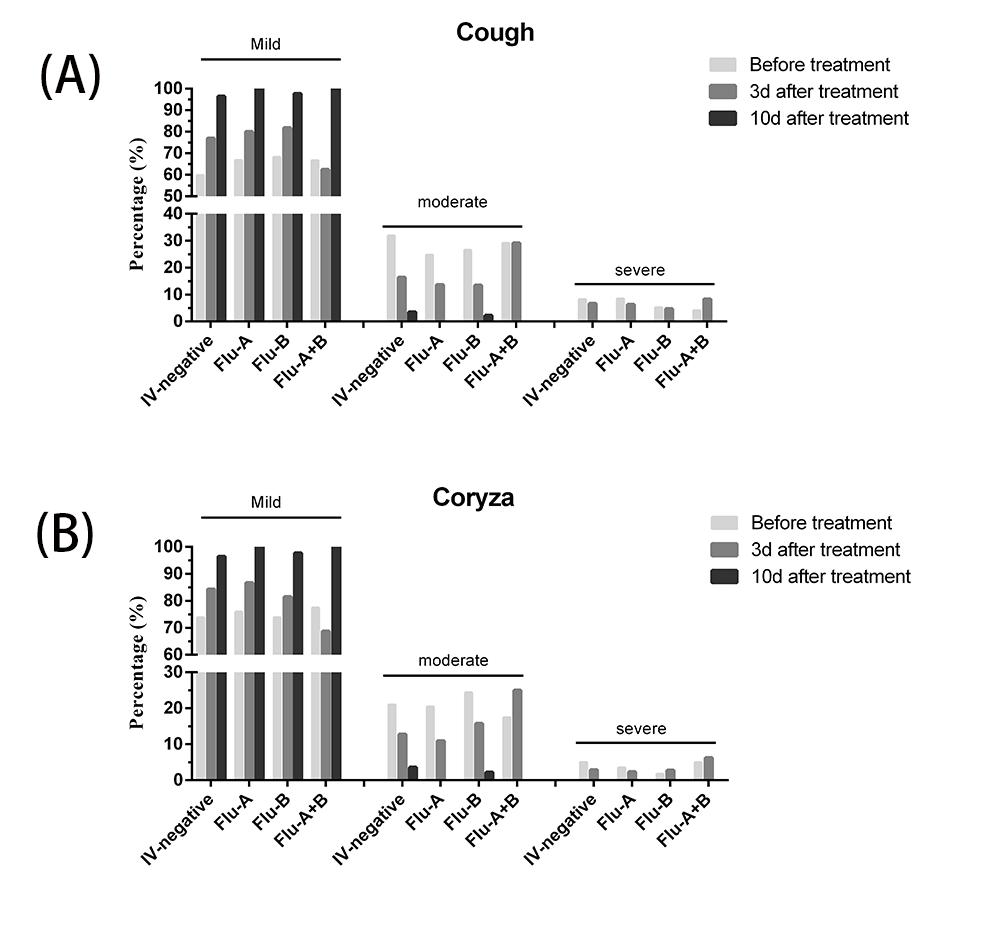

Supplement: Supplementary file 1 [file Image3.TIF]

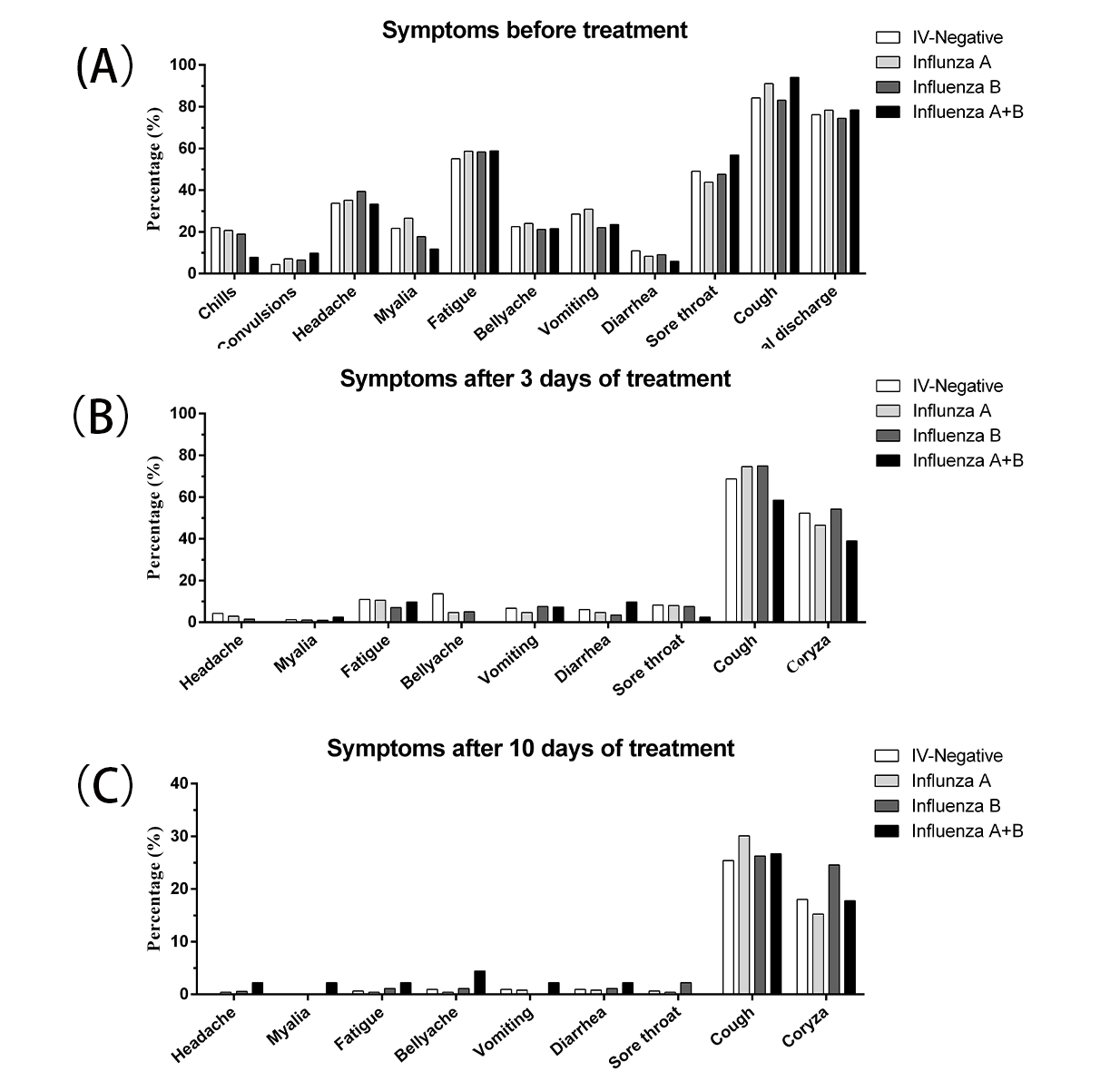

Supplement: Supplementary file 2 [file Image2.TIF]
